# Report of the Malignant Fever Called the "Pali Plague," Which Has Prevailed in Some Parts of Rajpootana, Since July 1836

**Published:** 1839-01-01

**Authors:** 


Report of the Malignant Fever, called the "Pali Plague," which has prevailed in some parts of Rajpootana, since the Month of July, 1836. By James Rankin, M.D. Published by authority of the Indian Government. Octavo, 1838, Calcutta, G. H. Huttman.

In a short notice which we took of the first volume of the Bombay Medical Transactions, we adverted to a kind of "Plague" which ravaged some of the upper provinces of India, in the years 1818 and 1819, but of which we heard little or nothing in this country till the present time. The volume before us draws our attention to a "plague" of still more recent date, and which will probably supersede the terrors of the cholera, since our Indian brethren seem to attach the character of contagion—or at least infection—much more to this epidemic (if the term be allowed) than to the famous plague of Jessore, of 1817. We shall endeavour to compress our notice of this volume into as narrow a compass as possible, well knowing the indifference of European readers to any thing Oriental which is not likely to come home to their own doors. They had better remember the cholera, however, which was much less likely to pay us a visit than the "Pali Plague" is, if the infectious nature of the malady is truly estimated.

It appears that, in the month of July 1836, a destructive fever broke out in the principality of Joudpoor, or Marwar, at Pali, a large town, which is reckoned the emporium of the trade between central India and the sea-ports of Guzerat. In that place 650 of the Chepahs (printers of plain cloth), died—then suffered the Brahmins—next, the retail merchants—and lastly, the inhabitants generally. It is supposed that out of a population of 15 or 20 thousand, four thousand died, at the rate of 50 or 60 daily. Many fled to the neighbouring villages, and the disease appears to have spread with them. In September the epidemic reached Joogit, and in October it invaded Goodpour, the capital of Marwar. Passing over a hilly tract, it invaded Deogurh in Meywar, and reached Rhambgur in the district of Ajmere. By April the sickness had approached the British cantonment, near Nusserabad, and then alarm was created. The mortality was rated by the inhabitants at one hundred thousand souls—but this was perhaps an exaggeration. Not more than one in three recovered. Mr. Maclean was despatched to Pali, to ascertain the nature of the malady, and he pronounced it to be the "plague"—"though not in its worst form." Dr. Irvine, also, came to the same conclusion, contrary to first impressions. Dr. A. Kier "did not dissent from his professional brethren." All, in short, who saw the malady, agreed that it was the PLAGUE.

The Bombay Government took the lead in precautionary measures, a similar disease having prevailed in some of these provinces in 1821.

"Happily without experience in meeting such a calamity, Sir Charles Metcalfe, seems to have taken for his guide Sir Thomas Maitland's regulations, and Mr. Tully's account of their practical efficacy, in excluding or extinguishing Plague in the Mediterranean Islands. The minute, based on this authority, consequently assumed the leading principle that the disease, being simply contagious, might be stopped and escaped by avoiding contact with the sick, or close proximity to them and what had touched their bodies. From this fundamental doctrine, very fully explained in that document, the instructions were deduced in detail: and a modification of arrangements which had proved so successful in insular situations, was, as far as it could be, made applicable to a continental country divided only by arbitrary boundaries. Exactly the same measures, however, seemed demanded for the protection of both localities when once the Plague had got admission into them. In anticipation of such an event, therefore, orders were given to isolate every town or village in our territory as soon as it became affected; and to allot six separate places within it to the following purposes: 1st—An hospital for the infected. 2nd—A depot for the strongly suspected. 3rd—Another depot for the slightly suspected. 4th—A place of quarantine for new comers. 5th—A depot for infected or suspected goods, where they might be deposited until purified; and 6th, a residence for expurgators. These expurgators conveying the sick to the hospital, and the dead to their graves, disinfecting their houses and destroying tainted articles, were to do all the duties expected of persons bearing that designation in the East of Europe. The line of circumvallation, guarded partly or wholly by armed men, to prevent ingress and egress, was to allow no provisions to enter unless by authority of the Quarantine Officers; and in paying for them, the inhabitants of the infected spot were to drop their money into a cup of water, from whence the sellers might take it without risk." 8.

Thus the beautiful code of quarantine promulgated by our first Cholera Board in London, and embellished in the Quarterly Review, was firmly established over the boundless hills, plains, jungles, and woods of India. The disease has hitherto confined itself (we beg pardon, *has been confined)* within the limits of Marwar and Merjar; but the following passage is somewhat startling.

"In finishing this brief history of the Pali disease, I have to mention with reference to the discussion which will be introduced in another section, that an *epidemic with the same pathognomonic symptoms* has been known in the mountainous territory of Kamaoon since 1823: that while the reputed Plague prevailed in the west, common intermittents and remittents existed in the intermediate space; and an infectious *yellow fever* devastated our villages and jails in the east, of Upper India." 12.

### DESCRIPTION.

"The symptoms of the Pali disease are those of other severe fevers common in India, with the superaddition of swellings of the external glands." "The presence of buboes and the absence of yellowness of the skin are the only remarkable circumstances which distinguish it from the Moradabad epidemic—both maladies having been, in an equal degree, infectious." The glandular affection is the sole peculiarity of the Pali disease, and as buboes are not the essential characteristic of the Levant plague, the Pali disease is not, therefore, plague in the sense of modern authors and teachers, implying its derivation, directly or indirectly from Egypt or the Levant—" but a fever which local causes produced, and circumstances rendered malignant and infectious." This is moderate doctrine—and may be very near the truth. The following concise symptomatology of the disease is interesting to us, even at this distance.

" No sense of indisposition gives warning of the approach of this disease. It comes on with slight rigor, headache, nausea, and pain in the loins. The skin soon grows hot and dry, the pulse, from 130 to 150 in the minute, is soft and easily compressible. The tongue is variously covered with white, light brown, or darkish fur, and these colours are sometimes intermixed. Vomiting and irritability of stomach occur, though rarely. The bowels are bound; the abdomen tumid and full, is seldom painful on pressure. The eyes appear heavy, maudlin and bloodshot, and occasionally look as if injected with lake. The countenance is expressive of anxiety and inward pain, the respiration apparently unaffected by the predominant malady, is often impeded by concomitant inflammation of the lungs. Glandular swellings appear in the groins, armpits, and neck, most frequently on the left side, as also under the jaws and ears, and in the upper part of the thigh. They are generally perceptible on the first or second day, and rarely encrease above the size of a walnut, but in some instances they become much larger, burst, and discharge purulent matter.

The symptoms are sometimes all so mild that the sick keep walking about till they gradually recover. In most of the cases observed there was a visible abatement of the disease every twenty-four hours, towards morning. But in the worst forms of it intense fever continued night and day without any remission, the patients could not rise from their cots on account of extreme debility, and an attempt to raise them to the erect posture produced fainting. Hemorrhage from the lungs took place in a few and was much dreaded. Delirium occurred rarely at the beginning, but coma generally supervened shortly before death. When violent the malady ran its fatal course in three days. When mild with or without the affection of the glands—it was protracted to fifteen or twenty days like the ordinary fevers of the country." 16.

Excepting then the glandular swellings, all the other morbid phenomena of the Pali Plague were observed in the contemporaneous fever raging from Kurnaul to Moradabad, as witnessed by numerous medical officers.

" All of them depict the same indications of pestilence, infection, prostration of strength, the muddy and blood-shot eye, the white crusted tongue, hemorrhage, delirium, coma, and rapid dissolution of two-thirds of those taken ill. This eastern epidemic of Upper India, with all, except one, of the signs of the western malady, had others which are less common in this country. It exhibited in particular that instantaneous and mortal depression apparently unpreceded by any morbid action or sensation, a phenomenon characteristic of the worst form of pestilence, which seems never to have occurred in Rajpootana. It is well known that the Greek word, from which the Plague derives its modern name, signifies a *stunning blow*.

But the most remarkable exception to the uniformity of the two disorders is that, at Pali the external glands, and at Moradabad the hepatic system, are prominently deranged. Hence buboes obtained for the more distant one the name with all the terrors of *Plague*, whilst the other, in the heart of our North Western Provinces, though jaundice attended it, being simply called *fever*, created no alarm and hardly attracted notice." 24.

Our author, while he believes the Pali Plague to be an infectious fever of local origin, similar to the Levant disease, gives up all idea of its being imported from the one country into the other. The sources of this epidemic are, with probability, traced to vegeto-animal exhalations from the surface of the soil.

The author has brought forward authentic facts, and has reasoned well in support of the foregoing opinion, and we quite coincide with him in the conclusions which he has drawn.

In respect to the treatment, so few opportunities were afforded to the European practitioners, that little more can be gleaned from their reports than that the means employed in fever generally, were those chiefly trusted to in the Pali Plague. We shall conclude with the following most sensible and judicious observations :—

"The fact that the Moradabad fever, though equally infectious and severe, attacked none but the poorer classes of natives in foul and ill-ventilated places, authorises a belief that had the Pali disease extended to the British provinces, it would have been confined to similar persons and localities.

Quarantine and preventive lines, by stopping some individuals, in whom the distemper lurked and who if taken ill subsequently might have caused it to spread in villages within our territory, like those in which the sickness originated, no doubt lessened the chances of the infection being carried beyond the cordons. Such precautionary measures are therefore so far beneficial. But suspending trade and labour on which the physical well-being of the people mainly depends, the good which they do is more than counterbalanced by their tendency to produce famine and the very diseases that they are intended to prevent or eradicate. The possibility of making extensive cordons impassable on continental frontiers, were it desirable, appears doubtful." 53.

A chart accompanies the work, in which are laid down the towns and villages in which prevailed three apparently different epidemic diseases—the Pali Plague—yellow fever—and the remittent fevers of India generally. That these were all three modifications of one and the same disease, there can be no rational doubt. In some localities the common remittent of hot climates was undisturbed in its course and features, or only aggravated in degree—in other localities the force of local causes fell on the hepatic system, and jaundice was the prominent feature—and other places, the characteristic feature displayed itself in the glandular system, and buboes, &c. reminded the spectators of the Plague of Egypt and the Levant.

In a large Appendix are recorded the reports and statements of several medical officers, from which documents the author has drawn up his deductions with great perspicuity, moderation, and ability.

